# Association of PM_2.5_ exposure with hospitalization for cardiovascular disease in elderly individuals in Japan

**DOI:** 10.1038/s41598-021-89290-5

**Published:** 2021-05-10

**Authors:** Toshiki Kaihara, Kihei Yoneyama, Michikazu Nakai, Takumi Higuma, Yoko Sumita, Yoshihiro Miyamoto, Mika Watanabe, Masaki Izumo, Yuki Ishibashi, Yasuhiro Tanabe, Tomoo Harada, Satoshi Yasuda, Hisao Ogawa, Yoshihiro J. Akashi

**Affiliations:** 1 Division of Cardiology, Department of Internal Medicine, St. Marianna University School of Medicine, 2-16-1, Sugao, Miyamae-ku, Kawasaki, Kanagawa 216-8511 Japan; 2 Department of Statistics and Data Analysis, Center for Cerebral and Cardiovascular Disease Information, National Cerebral and Cardiovascular Center, Suita, Osaka Japan; 3 Department of Cardiovascular Medicine, National Cerebral and Cardiovascular Center, Suita, Osaka Japan

**Keywords:** Cardiovascular biology, Epidemiology

## Abstract

Although exposure to particulate matter with aerodynamic diameters ≤ 2.5 µm (PM_2.5_) influences cardiovascular disease (CVD), its association with CVD-related hospitalizations of super-aged patients in Japan remains uncertain. We investigated the relationship between short-term PM_2.5_ exposure and CVD-related hospitalizations, lengths of hospital stays, and medical expenses. We analyzed the Japanese national database of patients with CVD (835,405) admitted to acute-care hospitals between 2012 and 2014. Patients with planned hospitalizations and those with missing PM_2.5_ exposure data were excluded. We classified the included patients into five quintiles based on their PM_2.5_ exposure: PM-5, -4, -3, -2, and -1 groups, in descending order of concentration. Compared with the PM-1 group, the other groups had higher hospitalization rates. The PM-3, -4, and -5 groups exhibited increased hospitalization durations and medical expenses, compared with the PM-1 group. Interestingly, the hospitalization period was longer for the ≥ 90-year-old group than for the ≤ 64-year-old group, yet the medical expenses were lower for the former group. Short-term PM_2.5_ exposure is associated with increased CVD-related hospitalizations, hospitalization durations, and medical expenses. The effects of incident CVDs were more marked in elderly than in younger patients. National PM_2.5_ concentrations should be reduced and the public should be aware of the risks.

## Introduction

Cardiovascular disease (CVD) is the leading cause of death due to non-communicable disease^1^. According to NHANES 2013 to 2016 data^2^, the CVD morbidity increases with age in both sexes, and the prevalence of coronary heart disease which is one of the major CVDs, is 6.7% in US adults. Particulate matter with aerodynamic diameters ≤ 2.5 µm (PM_2.5_) in the atmosphere increases cardiovascular morbidity and cardiovascular and overall mortality^3,4^ as well as the risk of developing CVDs, such as myocardial infarction^5^, heart failure^6^, arrhythmia^7^, hypertension^8^, and ischemic stroke^9^. According to the American Heart Association, the existing evidence supports a causal relationship between PM_2.5_ exposure and cardiovascular mortality and morbidity^10^.

Moreover, few previous studies have focused on the results for individuals > 90 years old. Japan is a super-aged society, with an average life expectancy of 80 years for men and 86 years for women^11^. Currently, there is a lack of clarity regarding whether CVDs in the super-aged societies of many countries are similarly affected by PM_2.5_ exposure and whether older people are more susceptible to such pollution than younger people. Most epidemiological studies focus on mortality data; thus, published studies have not examined the association between PM_2.5_ exposure and CVD-related health care costs, using Japanese nationwide databases. The aging of Japan’s population is not only resulting in a rapid population decline, it is also contributing to longer hospital stays and higher medical costs^12,13^.

To address these issues, we combined two nationwide databases. The Japanese Registry Of All cardiac and vascular Diseases (JROAD), which contains information on CVD diagnosis, hospitalization duration, and medical costs and the National Institute for Environmental Studies database includes data on environmental pollution. We conducted an observational study to investigate whether the prevalence of all CVDs and the related medical costs are related to short-term PM_2.5_ exposures.

## Results

In the included study population, the median patient age was 76 years, and the male/female distribution was 58%/42%. The percentages of cases with acute myocardial infarction, heart failure, aortic disease, pulmonary embolism, and cardiac arrest were 10.6%, 29.3%, 5.5%, 1.2%, and 9.0%, respectively. A comparison of patient characteristics between the included and excluded patients is shown in Supplementary Table S1.

### PM_2.5_ exposure and the CVD prevalence rate

Multilevel, mixed-effects Poisson regression (Table 1) revealed that the CVD prevalence rate was positively associated with an increase in PM_2.5_ exposure (prevalence ratio, 1.00071/1-μg/m^3^ of PM_2.5_; *p* < 0.001). Compared with that of the PM-1 group, the CVD-related hospitalization rate was higher for all the other groups (*p* < 0.001 for PM-5, -4, and -3; *p* = 0.025 for PM-2).

**Table 1:** Association between PM_2.5_ exposure and the CVD prevalence rate.

All (n = 835,405)			Multilevel, mixed-effects Poisson regression^a^	
			Prevalence ratio (95% CI)^b^	*p*
PM_2.5_ (continuous values)			1.00071 (1.00070–1.00072)	< .001
PM_2.5_ (categorical values)	Groups^c^	PM-1 (< 7.7 µg/m^3^)	Ref	
		PM-2 (7.7—11.0 µg/m^3^)	1.00024 (1.00003–1.00045)	0.025
		PM-3 (11.0—14.8 µg/m^3^)	1.00622 (1.00600–1.00643)	< .001
		PM-4 (14.8—20.3 µg/m^3^)	1.01955 (1.01933–1.01976)	< .001
		PM-5 (> 20.3 µg/m^3^)	1.02129 (1.02106–1.02152)	< .001
PM_2.5_ by age category	Age category	Age ≤ 64 y (n = 201,627)	1.00064 (1.00062–1.00066)	Ref
		Age 65–74 y (n = 178,850)	1.00076 (1.00074–1.00078)	< .001
		Age 75–89 y (n = 376,874)	1.00072 (1.00071–1.00073)	< .001
		Age ≧90 y (n = 78,054)	1.00082 (1.00079–1.00084)	< .001

CVD, cardiovascular disease; PM_2.5_, particulate matter with aerodynamic diameters ≤ 2.5 µg/m^3^; CI, confidence interval.

^a^The mixed models were used to correct for random effects due to interhospital variation.

^b^Prevalence ratios were adjusted for temperature, humidity, eastern/western Japan, season, number of hospital beds, age, sex, height, weight, Brinkman index, and Charlson comorbidity index.

^c^Classified according to PM_2.5_ exposure concentration.

There was an interaction between PM_2.5_ exposure and age regarding their effect on the CVD prevalence rate (*p* < 0.001). Compared with that of the ≤ 64-year group, the effect of PM_2.5_ exposure on the CVD-related hospitalization rate was greater for all other age groups (all, *p* < 0.001).

### PM_2.5_ exposure and length of hospital stay

**Figure 1:**
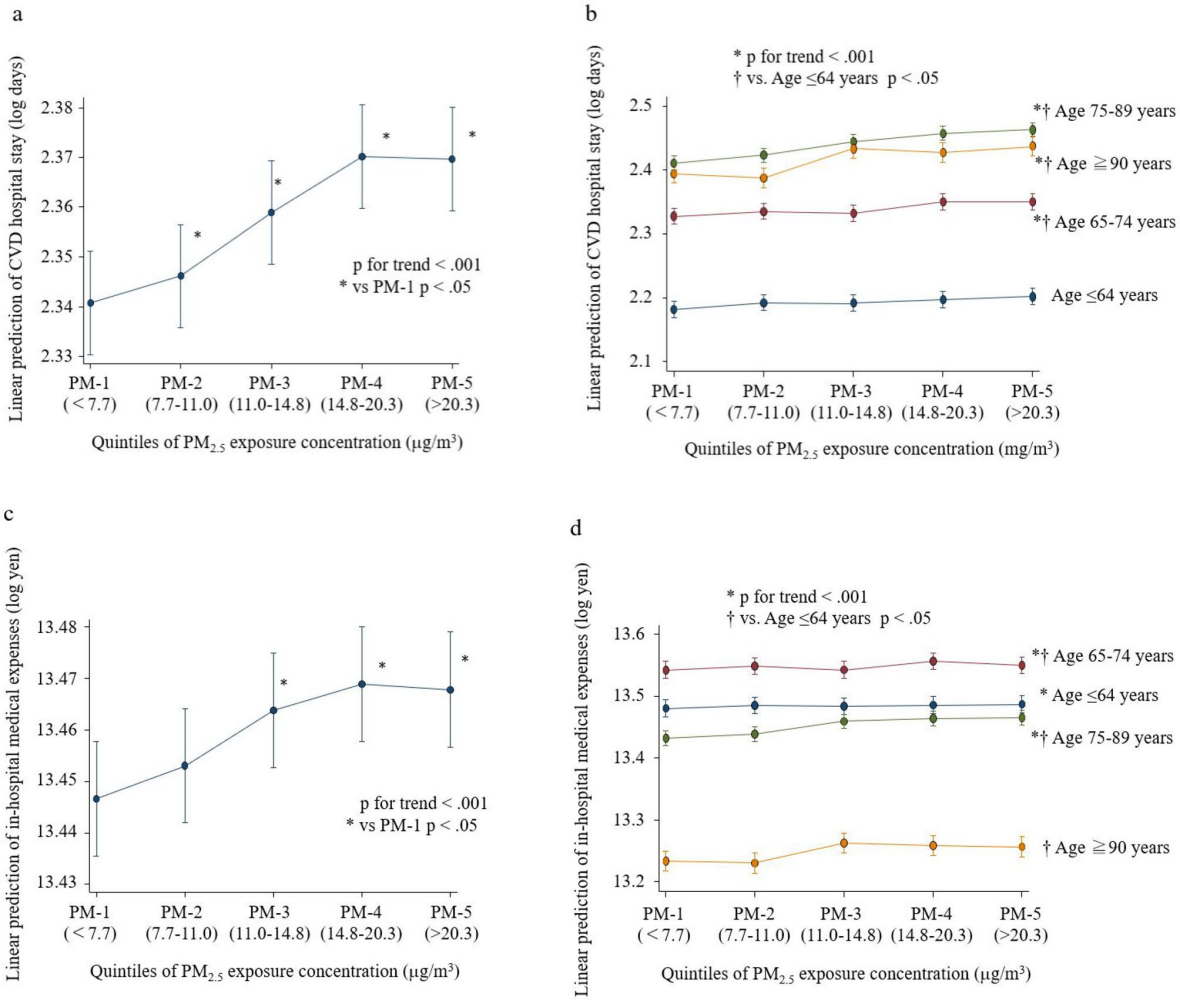
Relationship between PM_2.5_ exposure and length of CVD-related hospital stays and in-hospital medical expenses. The predicted lengths of CVD-related hospital stays and in-hospital medical expenses were calculated using the “marginsplot” command in StataCorp 2015. *Stata Statistical Software: Release 14*. College Station, TX: StataCorp LP., after the multilevel, mixed-effects, linear regression analyses were performed, as indicated in Tables 2 and 3. Error bars represent predictive margins with 95% confidence intervals. (**a**) The overall predicted length of hospital stays. (**b**) The predicted lengths of hospital stays per age group. (**c**) The overall predicted in-hospital medical expenses. (**d**) The predicted in-hospital medical expenses per age group. Each age group is indicated by a different colored line (≤ 64 years [blue], 65–74 years [red], 75–89 years [green], and ≧ 90 years [yellow]) in b and d.

Multilevel, mixed-effects linear regression (Table 2) demonstrated that the lengths of CVD-related hospital stays (log days) were positively associated with an increase in PM_2.5_ exposure (regression coefficient [95% confidence interval {CI}], 0.006 [0.005–0.008]/1-μg/m^3^ of PM_2.5_; *p* < 0.001) (Fig. 1a). Compared with that of the PM-1 group, the CVD-related hospital stays were longer for all other groups (*p* < 0.001 for PM-5, -4, and -3; *p* = 0.048 for PM-2).

**Table 2:** Association between PM_2.5_ exposure and lengths of CVD-related hospital stays (log days).

All (n = 835,405)			Multilevel, mixed-effects linear regression^a^	
			Regression coefficient (95% CI)^b^	*p*
PM_2.5_ (continuous values)			0.006 (0.005–0.008)	< .001
PM_2.5_ (categorical values)	Groups^c^	PM-1 (< 7.7 µg/m^3^)	Ref	
		PM-2 (7.7–11.0 µg/m^3^)	0.006 (< 0.001–0.011)	0.048
		PM-3 (11.0–14.8 µg/m^3^)	0.018 (0.013–0.024)	< .001
		PM-4 (14.8–20.3 µg/m^3^)	0.029 (0.024–0.035)	< .001
		PM-5 (> 20.3 µg/m^3^)	0.029 (0.023–0.035)	< .001

CVD, cardiovascular disease; PM_2.5_, particulate matter with aerodynamic diameters ≤ 2.5 µm; CI, confidence interval.

^a^The mixed model was used to correct for random effects due to interhospital variations. ^b^Regression coefficients were adjusted for temperature, humidity, eastern/western Japan, season, number of hospital beds, age, sex, height, weight, Brinkman index, Charlson comorbidity index, angina pectoris, acute myocardial infarction, heart failure, atrial fibrillation/flutter, aortic disease, cardiac arrest, pulmonary embolism, pulmonary hypertension, and tetralogy of Fallot. ^c^Classified according to PM_2.5_ exposure concentration.

There was no evident interaction between PM_2.5_ exposure and age in terms of lengths of hospital stay (*p* = 0.871). CVD-related hospital stays were shorter for the ≤ 64-year group than for any other age group (all, *p* < 0.05) (Fig. 1b). There was a positive relationship between PM_2.5_ exposure and lengths of CVD-related hospital stays for all age groups, except the ≤ 64-year age group (*p* for trend, < 0.001).

### PM_2.5_ exposure and in-hospital medical expenses

Multilevel, mixed-effects, linear regression analysis (Table 3) indicated that in-hospital medical expenses (log yen) were positively associated with an increase in PM_2.5_ exposure (regression coefficient [95% CI], 0.008 [0.007–0.009]/1-μg/m^3^ of PM_2.5_; *p* < 0.001) (Fig. 1c). Compared with those of the PM-1 group, the in-hospital medical expenses were higher in three of the other groups (*p* < 0.001 for PM-5, -4, and -3).

**Table 3:** Association between PM_2.5_ exposure and in-hospital medical expenses (log yen).

All (n = 835,405)			Multilevel, mixed-effects linear regression^a^	
			Regression coefficient (95% CI)^b^	*p*
PM_2.5_ (continuous values)			0.008 (0.007–0.009)	< .001
PM_2.5_ (categorical values)	Groups^c^	PM-1 (< 7.7 µg/m^3^)	Ref	
		PM-2 (7.7—11.0 µg/m^3^)	0.0036 (− 0.0021–0.0094)	0.21
		PM-3 (11.0—14.8 µg/m^3^)	0.016 (0.010–0.022)	< .001
		PM-4 (14.8—20.3 µg/m^3^)	0.023 (0.017–0.029)	< .001
		PM-5 (> 20.3 µg/m^3^)	0.021 (0.016–0.027)	< .001

PM_2.5_, particulate matter with aerodynamic diameters ≤ 2.5 µg/m^3^; CI, confidence interval.

^a^The mixed model was used to correct for random effects due to interhospital variations.

^b^Regression coefficients were adjusted for temperature, humidity, eastern/western Japan, season, number of hospital beds, age, sex, height, weight, Brinkman index, Charlson comorbidity index, angina pectoris, acute myocardial infarction, heart failure, atrial fibrillation/flutter, aortic disease, cardiac arrest, pulmonary embolism, pulmonary hypertension, and tetralogy of Fallot.

^c^Classified according to PM_2.5_ exposure concentration.

There was no interaction between PM_2.5_ exposure and age in terms of in-hospital medical expenses (*p* = 0.371). In-hospital medical expenses in the ≤ 64-year group were lower than those in the 65–74-y group and higher than those in the 75–89-year and ≥ 90-year groups (all, *p* < 0.05) (Fig. 1d). PM_2.5_ exposures and in-hospital medical expenses were positively correlated in all age groups, except the ≥ 90-year group (*p* for trend, < 0.001).

The specific risk estimates for the CVD prevalence rate on PM_2.5_ exposure are summarized in Supplementary Fig. S1. There was an interaction between PM_2.5_ exposure and age in terms of the effect on the CVD prevalence rate (*p* < 0.001). Compared with that of the ≤ 64-year group, the effect of PM_2.5_ exposure on the CVD-related hospitalization rate was greater for all other age groups (all, *p* < 0.001). The effect was greater in males; those with angina, acute myocardial infarction, heart failure, aortic disease, pulmonary embolism, atrial fibrillation or flutter, cardiac arrest; those in east Japan; and on weekdays (all, *p* < 0.001). The effect was also greater in the summer and winter than in the spring (both, *p* < 0.001), but contrarily, was less in the autumn (*p* < 0.001).

## Discussion

We conducted an observational study using a Japanese nationwide registry database to investigate the effects of PM_2.5_ exposure on CVD-related hospitalizations. The outcomes of the study may be summarized as three main findings. (1) Short-term PM_2.5_ exposure is positively related to the prevalence rate of hospitalizations due to CVDs, particularly among the elderly. (2) Short-term PM_2.5_ exposure is positively associated with the lengths of CVD-related hospitalizations; hospitalizations tended to be longer for elderly patients than for younger patients. (3) Short-term PM_2.5_ exposure is positively related to in-hospital medical expenses, and medical expenses tend to be lowest for patients ≥ 90 years old (Fig. 1). These observations suggest that lowering PM_2.5_ exposures will reduce not only the risk of CVD development but also the lengths of hospital stays and medical expenses in a super-aged society.

The mechanism of the association between PM_2.5_ exposure and CVDs has been discussed extensively. Pope et al.^14^ discovered that acute PM_2.5_ exposure is associated with increased endothelial cell apoptosis and systemic inflammation in healthy young adults. Magari et al.^15^ observed a negative relationship between PM_2.5_ exposure and heart rate variability in the working population. Huang et al.^16^ indicated that seven days of exposure to high concentrations of PM_2.5_ is associated with substantial blood pressure increases. In addition to these reported mechanisms, the American Heart Association stated that short-term PM_2.5_ exposure is related to elevations in inflammatory markers, like C-reactive protein, systemic oxidative stress (negative biological impact on cells or tissues), thrombosis (impacts coagulation pathway), and epigenetic changes (subclinical pathophysiological responses)^10^. Additionally, a recent meta-analysis of PM_2.5_ exposure and all-cause mortality demonstrated increased risks of all-cause and cause-specific mortality related to short-term PM_2.5_ exposures, consistent with above-mentioned findings^17^. Further, Bonzini et al.^18^ observed that long-term PM_2.5_ exposure levels were associated with decreased prothrombin times, elevated endogenous thrombin potentials, and elevated C‐reactive protein concentrations in steel production workers. Ljungman et al.^19^ revealed that a 2-h increase in PM_2.5_ triggered ventricular arrhythmias in patients with implantable cardioverter-defibrillators. Thus, short-term PM_2.5_ exposures may cause endothelial dysfunction, increased oxidative stress, and inflammation, all of which may lead to CVDs^20^.

Of note, our study showed that the effects of PM_2.5_ exposure were more pronounced in elderly patients than in younger patients. The exact mechanism for this is not clear; however, the long-term effects of PM_2.5_ exposure include atherosclerosis. In large, population-based cohort studies of adults without pre-existing CVDs, long-term PM_2.5_ exposure was associated with incident hypertension^21^, retinal arteriolar narrowing^22^, coronary calcification^23^, thoracic aortic calcification^24^, and intima-media thickening^25,26^. These risk factors are also known to be associated with age and the development of CVDs, suggesting that short-term PM_2.5_ exposures may be the triggers associated with the higher risks of CVDs in the elderly, compared with younger people. The relationship between PM_2.5_ exposures and the development of the specific CVDs shown in the present study (Fig. S1) is compatible with this suggestion.

Non-CVD-related complications, such as organ damage, may explain why PM_2.5_ exposure prolongs hospital stays and increases medical expenses. For example, PM_2.5_ exposure is known to be associated with other underlying medical problems, such as pulmonary oxidative stress and disorders of the vasculature, blood flow, liver metabolism, nervous system, etc^9,10,13,27^.

An analysis of “big data” in a U.S. study of PM_2.5_ exposures (95,277,269 inpatient hospital claims between 2000 and 2012) demonstrated that each 1-µg/m^3^ increase in short-term PM_2.5_ exposure was associated with an annual increase of 20,098 hospitalization days (95% CI, 18,950–21,247 days) and 69 million USD (95% CI, 65–73 million USD) in inpatient and post-acute-care costs^28^. Our big-data analysis revealed a similar trend. An unexpected finding from our study was that the in-hospital medical costs of patients ≥ 90 years old were not positively related to increasing PM_2.5_ exposures, despite showing a positive relationship with the lengths of hospital stays. Although we did not investigate the reasons for this finding, Japan’s policy of not performing high-cost procedures on patients ≥ 90 years may contribute to this unexpected relationship.

PM_2.5_ exposure is not only a problem in Japan, but also in neighboring countries. CVDs are a growing public health concern in all countries with aging populations. Therefore, the findings of the present study may have important implications for CVD risk reduction and related health care costs in similar populations. Further, these results may guide public health interventions that minimize cardiovascular effects due to PM_2.5_ exposure.

The main strengths of our study are its large sample size and its national scale. Thus, the findings are expected to reflect the actual effects of PM_2.5_ exposures throughout Japan. Furthermore, we separately analyzed the data for patients ≥ 90 years old. One study limitation is that the data were obtained only from DPC hospitals with cardiovascular care facilities that meet the Japanese Circulation Society requirements (the characteristics of DPC hospitals were previously reported^29^). However, the JROAD is the largest database of nationwide cardiac health outcomes in Japan. Another limitation was that PM_2.5_ levels were measured at the local weather station closest to the hospital where the patient was admitted, not at the patient’s home address. Additionally, PM_2.5_ levels were not measured in the same way at each weather station. For example, the dataset does not include the altitude at which the measurements were made nor the sampling location’s proximity to a road. Therefore, some degree of potential exposure misclassification was unavoidable. Although we obtained the data from publicly available data observed by the National Institute for Environmental Studies, lack of detailed information on PM_2.5_ measurement may have biased our results towards the null. Further, we did not conduct specific analyses to distinguish between urban and rural areas or account for the socioeconomic status of each area; thus, we were unable to control for these potentially confounding factors. Moreover, the effect size of PM_2.5_ exposure on CVDs in this study was very small as we divided the number of CVDs admitted to DPC hospitals by the national population size. In addition, we only evaluated the effects of short-term PM_2.5_ exposures. Therefore, some CVD-related hospitalizations may be attributable to a “harvesting effect,” wherein the impending morbidity of the most vulnerable individuals in the population may merely have moved forward in time after a short-term PM_2.5_ exposure. This might be similar to one of the ischemic heart disease mortality conclusions from a published time series^30^ that “excluding short-term changes definitely lead to an increase in the estimated effect of air pollution.” Lastly, we showed the seasonal differences in CVD prevalence rates in Fig. S1; spring and autumn had negative impacts and summer and winter had positive impacts on the CVD prevalence rates associated with PM_2.5_ exposures. We plan to perform further investigations regarding these associations.

## Conclusions

Our big-data analysis of Japan’s super-aged society demonstrated that PM_2.5_ exposure is associated not only with cardiovascular events but also with the lengths of CVD-related hospital stays and in-hospital medical expenses. Furthermore, the effects were more pronounced in elderly patients than in younger ones. Our findings suggest that reducing the burden of CVD-related diseases and their associated medical costs may require reducing the national PM_2.5_ concentrations. Increasing the public's awareness of the dangers of PM_2.5_ exposure is important.

## Methods

### Data collection

We collected the data for all acute-care hospital admissions due to any type of CVD from the JROAD. The JROAD is a nationwide, prospective registry designed to assess the clinical activity of Japanese hospitals that provide cardiovascular care and provide information for improving each hospital’s patient care^31^. The Japanese Circulation Society created both the JROAD, which includes each hospital's demographics since 2004, and the JROAD-DPC, which includes data from the Japanese Diagnosis Procedure Combination/Per-Diem Payment System (DPC/PDPS) since 2014.

The DPC database is a case-mix classification system linked with a lump-sum payment system, launched in 2002 by the Ministry of Health, Labour and Welfare of Japan^31–36^; academic, urban, and rural hospitals use the DPC system. All data analyzed in the present study were from patients hospitalized because of clinically apparent CVDs. The DPC database is based on the International Classification of Disease (ICD) code of the illness for which the most medical resources were invested and which represents the cause of hospitalization, including CVDs. Doctors registered the ICD codes of the illness when the patients were discharged. Since ICD codes are divided into acute and chronic diseases, we could differentiate between acute and scheduled admissions in the DPC database.

PM_2.5_ and weather data were obtained from the database of the National Institute for Environmental Studies^37^. We used hourly temperature and humidity data and calculated the average daily temperature and humidity values. We also noted the PM_2.5_ concentrations and weather values for the day preceding each patient’s emergency hospitalization due to CVD. The localized weather and air pollution data were obtained by identifying the weather station closest to each hospital. We searched the weather station sites using the municipality code specifically assigned to each municipality by the Ministry of Internal Affairs and Communications; this allowed us to merge the hospital sites with the monitoring stations closest to them. We used the previous day’s data since data from the same day would have included post-admission data because the exact time of each patient’s admission is not available in the database. Thus, the PM_2.5_ exposure data in this study reflects only the patients’ short-term exposures.

**Figure 2:**
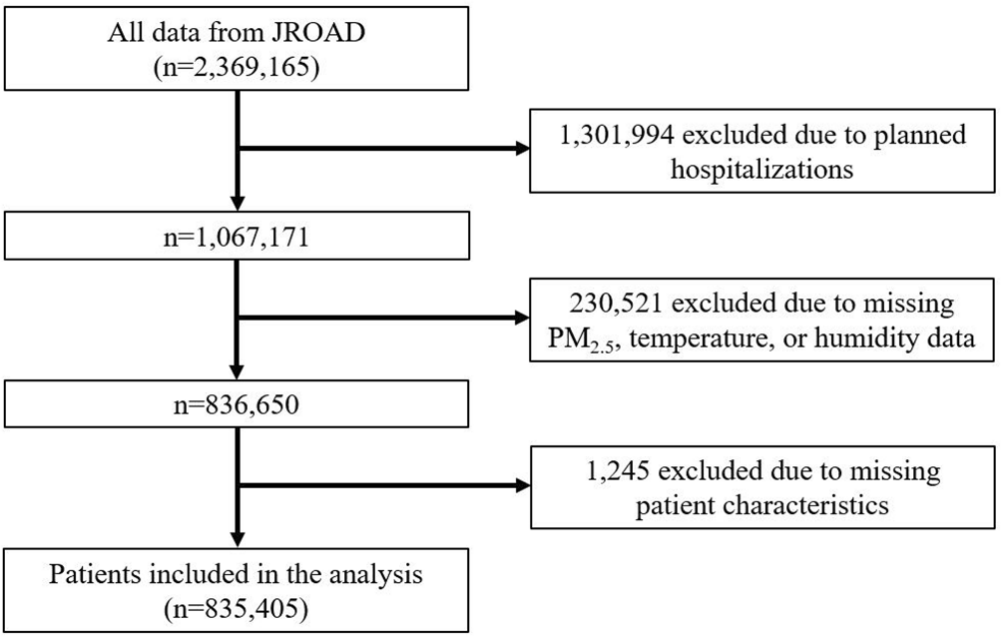
Study flow chart. This study included a large data set of patients from the Japanese Registry Of All cardiac and vascular Diseases (JROAD) obtained through the Japanese administrative case-mix Diagnostic Procedure Combination (DPC) system.

We conducted a cross-sectional study of the relationship between PM_2.5_ concentrations and CVD prevalence for the period between April 2012 and March 2014. We included 911 hospitals, where 2,369,165 consecutive patients were admitted during the study period. Patients were excluded due to: planned hospitalizations; missing PM_2.5_, temperature, or humidity data; or missing patient-characteristic data. Finally, 835,405 patients were included in the analysis (Fig. 2). Because the JROAD is not based on the numbers of patients but on DPCs, repeat admissions were considered as separate admissions. We classified the included patients into the following five groups, based on the ambient PM_2.5_ concentrations at the location where they were admitted: PM-5 (> 20.3 µg/m^3^), PM-4 (14.8–20.3 µg/m^3^), PM-3 (11.0–14.8 µg/m^3^), PM-2 (7.7–11.0 µg/m^3^), and PM-1 (< 7.7 µg/m^3^). We included analyses using PM_2.5_ as a continuous exposure in the supplementary materials (Supplementary Tables S2-4). The multivariable regression spline models (MVRS) command in Stata Statistical Software helped select the RS model that best predicts the outcome variables. MVRS analysis revealed a linear relationship between PM_2.5_ concentrations and some knots. The Japanese environmental standard for PM_2.5_ exposure is < 15 µg/m^3^ per day when averaged over a year, and < 35 µg/m^3^ as the maximum daily level.

This study design was approved by the Ethics Committee of St. Marianna University School of Medicine (No. 4038); the requirement for individual informed consent was waived because the data were anonymized when provided by the DPC. Further, this study was approved by the institutional review boards of all participating institutions and complies with the Declaration of Helsinki.

### Outcomes

The primary outcome was the prevalence of CVDs requiring hospitalization. CVDs were defined to include coronary artery disease, heart failure, arrhythmia, aortic or peripheral artery disease, cardiac arrest, pulmonary embolism, pulmonary hypertension, and endocarditis/pericarditis. The prevalence in this study refers to the number of CVD patients admitted per day. For the 900 CVD patients who were admitted on 15/5/2012, the prevalence was 900. For the 800 CVD patients who were admitted on 16/5/2012, the prevalence was 800. The prevalence (daily admissions) was calculated daily for two years. Other outcomes were per patient hospitalization durations and associated medical expenses.

### Statistical analyses

Patient characteristics are presented as medians and interquartile ranges for continuous values; categorical values are shown as frequencies and percentages. Wilcoxon’s rank-sum and chi-square test were used to compare the characteristics between included and excluded patients, in this study. A mixed, random-effects, population-averaged Poisson model was used to analyze the association between the number of CVD-related hospitalizations and PM_2.5_ exposures. The random effects were specified as the hospitals (institutions), using municipality codes to adjust for regional heterogeneity. Factors, such as hospital, weather, and patient factors were expected to contribute to the development of CVD. We modeled the association of PM_2.5_ concentrations with hospital data (eastern/western Japan; number of hospital beds; presence of coronary care unit[s], cardiac surgery service, and/or board-certificated cardiologists), weather data (season [Spring, March–May; Summer, June–August; Autumn, September–November; Winter, December–February], average temperature, and humidity), and patient demographics (age, sex, height, weight, Brinkman index^38^, and Charlson comorbidity index^39^). The Brinkman index is calculated as the product of the number of cigarettes smoked per day and the number of years of smoking. The Charlson comorbidity index is a score based on the presence or absence of 19 diseases that can influence mortality (congestive heart failure, chronic pulmonary disease, leukemia, metastatic tumor, etc.). These covariates thought to be related to the risk of developing CVD were carefully selected by experienced cardiologists. For the subgroup analysis, we classified the patients’ age into four groups according to the Japan Geriatrics Society guidelines^40^.

A mixed, random-effects, population-averaged linear model was used to analyze the associations of lengths of hospital stays and in-hospital medical expenses with PM_2.5_ exposures. The random effects were specified as the lengths of hospital stays and the in-hospital medical expenses. We modeled the association of PM_2.5_ exposure with hospital data, weather data, patient demographics, and CVD type (angina pectoris, acute myocardial infarction, heart failure, atrial fibrillation/flutter, aortic disease, cardiac arrest, pulmonary embolism, pulmonary hypertension, and tetralogy of Fallot). We included the CVD types in this model because in-hospital medical expenses and lengths of hospital stay may vary according to the CVD type. All analyses were performed in, and all graphs were constructed using, *Stata Statistical Software: Release 14*. (StataCorp LLC. 2015. College Station, TX, U.S.A.)

## Supplementary Information

Supplementary Information.

## Data Availability

The data that support the findings of this study are available from the JROAD, but restrictions apply to their availability; the data that were used were approved for use in the current study and are not publicly available. Data are, however, available from the JROAD upon reasonable request. Environmental pollution data are available from National Institute for Environmental Studies, Japan at http://www.nies.go.jp/db/index-e.html.
