# Supplementary material for: Association of PM2.5 exposure with hospitalization for cardiovascular disease in elderly individuals in Japan
Source: Sci Rep. 2021 May 10;11:9897. doi: 10.1038/s41598-021-89290-5 (PMC8110517; doi:10.1038/s41598-021-89290-5)
Supplement: Supplementary file 1 — Supplementary Information. [file 41598_2021_89290_MOESM1_ESM.pdf]

**SUPPLEMENTARY MATERIALS**

# Association of $PM_{2.5}$ exposure with hospitalization for cardiovascular disease in elderly individuals in Japan

Toshiki Kaihara, PhD, Kihei Yoneyama, PhD, Michikazu Nakai, PhD, Takumi Higuma, PhD, Yoko Sumita, Yoshihiro Miyamoto, PhD, Mika Watanabe, MD, Masaki Izumo, PhD, Yuki Ishibashi, PhD, Yasuhiro Tanabe, PhD, Tomoo Harada, PhD, Satoshi Yasuda, PhD, Hisao Ogawa, PhD, and Yoshihiro J. Akashi, PhD

**List**

- Supplementary Table S1: Patient characteristics
- Supplementary Table S2: Association between $PM_{2.5}$ exposure and the CVD incident rate
- Supplementary Table S3: Association between $PM_{2.5}$ exposure and length of CVD-related hospital stay (log days)
- Supplementary Table S4: Association between $PM_{2.5}$ exposure and in-hospital medical expenses (log yen)
- Supplementary Figure S1: Specific risk estimates of the CVD prevalence rate upon

$PM_{2.5}$ exposure

**Table S1. Patient characteristics**

| | Included patients (n=835,405) | Excluded patients (n=1,533,760) | p |
|---|---|---|---|
| Age, median (IQR) | 76 (65–84) | 77 (65–85) | < .001 |
| Males, n (%) | 484,003 (57.9) | 130,605 (57.0) | < .001 |
| ***Cardiovascular disease*** | | | |
| Angina pectoris, n (%) | 86,824 (10.4) | 23,261 (10.1) | < .001 |
| Acute myocardial infarction, n (%) | 88,269 (10.6) | 24,170 (10.5) | < .001 |
| Unstable angina, n (%) | 45,339 (5.4) | 11,591 (5.1) | < .001 |
| Atrial fibrillation or flutter, n (%) | 26,274 (3.1) | 7,525 (3.3) | < .001 |
| Heart failure, n (%) | 245,008 (29.3) | 68,437 (29.8) | < .001 |
| Aortic disease, n (%) | 46,204 (5.5) | 12,738 (5.6) | < .001 |
| Pulmonary embolism, n (%) | 10,037 (1.2) | 2,672 (1.2) | < .001 |
| Cardiac arrest, n (%) | 75,428 (9.0) | 16,108 (7.0) | < .001 |
| ***Coexisting conditions*** | | | |
| Charlson comorbidity index, median (IQR) | 1.0 (1.0–2.0) | 1.0 (1.0–2.0) | < .001 |
| Hospital stay, days (median [IQR]) | 12.0 (5.0–22.0) | 13.0 (6.0–23.0) | < .001 |
| Direct costs, yen (median [IQR]) | 767,041 (350,536–1,556,170) | 747,415 (348,377–1,500,000) | < .001 |

IQR, interquartile range

**Table S2. Association between $PM_{2.5}$ exposure and the CVD incident rate**

| All (n=835,405) | | Multilevel, mixed-effects Poisson regression[a] | |
|---|---|---|---|
| | | Prevalence ratio (95% CI)[b] | p |
| $PM_{2.5}$ (continuous values[c]) | $PM_{2.5}$ (<8.52) | 1.00618 (1.00610–1.00625) | < .001 |
| | $PM_{2.5}$ (8.52–12.83) | 1.00673 (1.00666–1.00680) | < .001 |
| | $PM_{2.5}$ (12.83–18.63) | 1.00444 (1.00437–1.00451) | < .001 |
| | $PM_{2.5}$ (>18.63) | 1.00072 (1.00066–1.00079) | < .001 |

[a]The mixed models were used to correct for random effects due to interhospital variation. [b]Prevalence ratios were adjusted for temperature, humidity, eastern/western Japan, season, number of hospital beds, age, sex, height, weight, Brinkman index, and Charlson comorbidity index.

[c]Classified according to three knots (8.52, 12.83, 18.63) accessed by multivariable regression spline models command in Stata.

CVD, cardiovascular disease; $PM_{2.5}$, particulate matter with an aerodynamic diameter ≤2.5 mm; CI, confidence interval.

We used the multivariable regression spline models (MVRS) command in Stata, which selects the RS model that best predicts the outcome variables. MVRS indicated a linear relationship with $PM_{2.5}$ concentrations, with three knots (8.52, 12.83, 18.63). The quintile of the original manuscript and the MVRS knots were almost the same. The table shows the prevalence ratio divided by knots.

**Table S3. Association between $PM_{2.5}$ exposure and length of CVD-related hospital stay (log days)**

| All (n=835,405) | | Multilevel, mixed-effects linear regression[a] | |
|---|---|---|---|
| | | Regression coefficient (95% CI)[b] | p |
| $PM_{2.5}$ (continuous values[c]) | $PM_{2.5}$ (<18.62) | 0.0024 (0.0020–0.0028) | < .001 |
| | $PM_{2.5}$ (>18.62) | -0.0001 (-0.0004–0.0004) | 0.908 |

[a]The mixed models was used to correct for random effects due to interhospital variation. [b]Regression coefficients were adjusted for temperature, humidity, eastern/western Japan, season, number of hospital beds, age, sex, height, weight, Brinkman index, Charlson comorbidity index, angina pectoris, acute myocardial infarction, heart failure, atrial fibrillation/flutter, aortic disease, cardiac arrest, pulmonary embolism, pulmonary hypertension, and tetralogy of Fallot.
[c]Classified according to one knot (18.62) accessed by multivariable regression spline models command in Stata.
CVD, cardiovascular disease; $PM_{2.5}$, particulate matter with an aerodynamic diameter ≤2.5 mm; CI, confidence interval.

The multivariable regression spline models indicated a linear relationship with $PM_{2.5}$ concentrations, with one knot (18.62), for hospital stays. This result and the original analysis were similar, so the original results were maintained.

**Table S4. Association between $PM_{2.5}$ exposure and in-hospital medical expenses**

**(log yen)**

| **All (n=835,405)** | | **Multilevel, mixed-effects linear regression[a]** | |
|---|---|---|---|
| | | **Regression coefficient (95% CI)[b]** | **p** |
| $PM_{2.5}$ (continuous values[c]) | $PM_{2.5}$ (<8.54) | 0.008 (0.006–0.01) | 0.029 |
| | $PM_{2.5}$ (8.54–18.63) | 0.005 (0.003–0.006) | < .001 |
| | $PM_{2.5}$ (>18.63) | -0.001 (-0.001–0.001) | 0.484 |

[a]The mixed model was used to correct for random effects due to interhospital variation.

[b]Regression coefficients were adjusted for temperature, humidity, eastern/western Japan, season, number of hospital beds, age, sex, height, weight, Brinkman index, and Charlson comorbidity index, angina pectoris, acute myocardial infarction, heart failure, atrial fibrillation/flutter, aortic disease, cardiac arrest, pulmonary embolism, pulmonary hypertension, and tetralogy of Fallot.

[c]Classified according to two knots (8.542, 18.630) accessed by multivariable regression spline models command in Stata.

$PM_{2.5}$, particulate matter with an aerodynamic diameter ≤2.5 mm; CI, confidence interval.

Regarding medical expenses, the multivariable regression spline models indicated a linear relationship with $PM_{2.5}$ concentrations, with two knots (8.542, 18.63). Because the result and the original analysis result was similar, we chose to maintain the original results.

**Figure S1. Specific CVD prevalence rate risk estimates for $PM_{2.5}$ exposure**

A forest plot for $PM_{2.5}$ exposure and CVD risk factors is shown. Adjusted prevalence ratios (95% CI) are described by black circles (black lines and boxes). The p-values for the prevalence ratios, by CVD risk factors, are shown. The p-values for the interactions of each factor are also shown.

CVD, cardiovascular disease; CI, confidence interval.

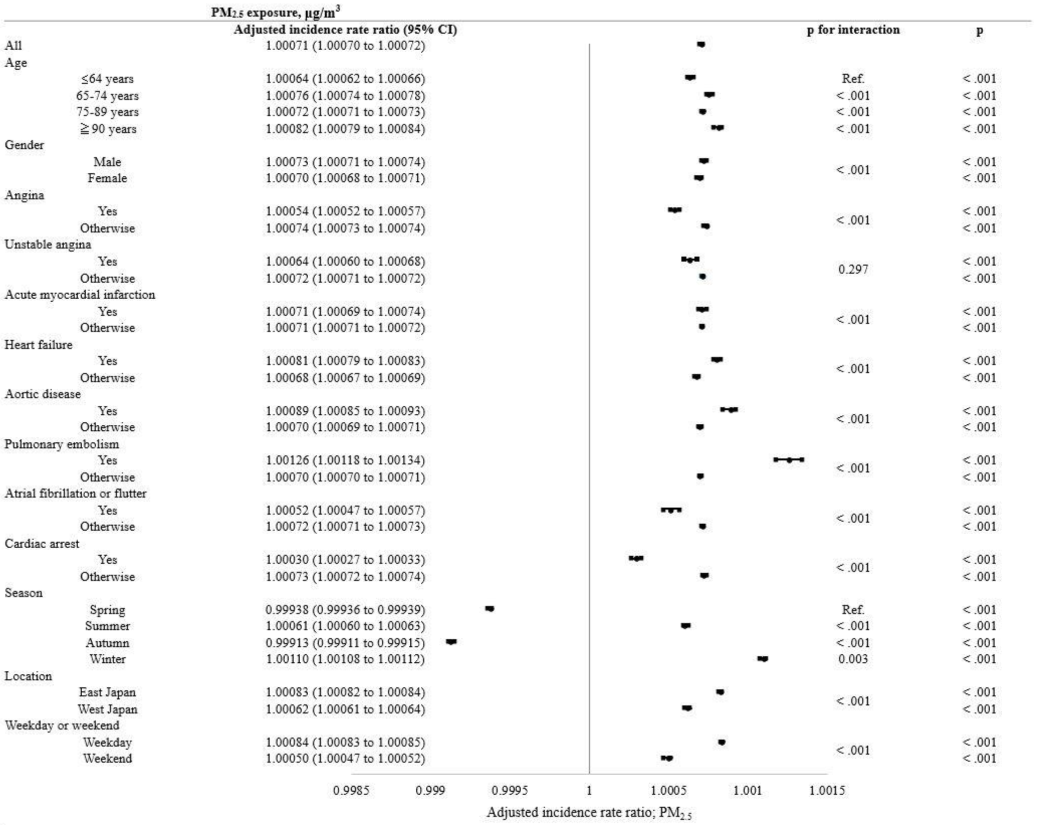


**Figure S1.**
